# The COVID-19 Pandemic in Care Homes: An Exploration of Its Impact across Regions in Spain

**DOI:** 10.3390/ijerph19159617

**Published:** 2022-08-04

**Authors:** Marta Benet, Patricia Celi-Medina, Montserrat Fernández, Sandra Ezquerra

**Affiliations:** 1 Campus Docent Sant Joan de Déu, School of Nursing, Universitat de Barcelona, Carrer Sant Benito Menni, 18-20, 08830 Sant Boi del Llobregat, Barcelona, Spain; 2 Research Group on Inclusive Societies, Politics, and Communities, Universitat de Vic-Universitat Central de Catalunya, Masia Torre dels Frares, Carrer Perot Rocaguinarda, 17, 08500 Vic, Barcelona, Spain; 3 UNESCO Chair on Women, Development, and Cultures, Universitat de Vic-Universitat Central de Catalunya, Masia Torre dels Frares, Carrer Perot Rocaguinarda, 17, 08500 Vic, Barcelona, Spain; 4 Department of Social Sciences and Community Health, School of Health and Welfare, Universitat de Vic-Universitat Central de Catalunya, Masia Torre dels Frares, Carrer Perot Rocaguinarda, 17, 08500 Vic, Barcelona, Spain

**Keywords:** COVID-19 pandemic, older adults, long-term care, homes for the aged

## Abstract

This article provides an updated picture of the enormous consequences that the first wave of the COVID-19 pandemic (March–June 2020) had for older adults living in Spanish care homes. It aims to describe the regional variation in deaths among home care residents through a methodological triangulation of descriptive quantitative, ecological and documentary analysis. Figures of five different indicators of care home mortality are provided and some factors related to higher mortality rates are presented and analysed in the descriptive ecological analysis in order to depict trends and, in a linear regression, to determine their statistical significance. The clearest trend reflected by the data is that the higher the cumulative incidence and the number of care home beds in the surrounding area, the higher the COVID-19 care home mortality. We argue that the pandemic has brought to light the historical fragility and underdevelopment of the Spanish LTC sector, and that these factors have exacerbated the consequences of the pandemic. Finally, we conclude that publicly available and disaggregated data would allow a deeper and more accurate analysis of potentially explanatory factors such as the type of care home ownership and management, and that further qualitative research would shed more light on people’s experiences.

## 1. Introduction

Since December 2019 the world has suffered the highly infectious and lethal impact of COVID-19, with millions of people falling ill (547,901,157) and 6,339,899 deceased [1] as of early July 2022. Older adults, especially those living in long-term care facilities, have been particularly at risk of severe COVID-19 disease and, as a result, they have experienced tragically high mortality rates [2,3,4]. Among the several risk factors for severe COVID-19 disease described in the literature, advanced age is the most relevant and widely discussed [5,6]. Epidemiological data suggest that the risk of serious complications, the need for both invasive mechanical ventilation (IMV) and transfer to ICU, the risk of in-hospital death and the mortality rate increase with age. In fact, most COVID-19-related deaths have occurred among people over 60, with the pandemic taking its greatest toll among those with chronic conditions related to cardiovascular or respiratory diseases [7,8]. According to World Health Organization (WHO) data, 30,098 people had died from COVID-19 in the European Region by April 2020. Over 95% of the deceased were over 60 and more than half of them were 80 or older [9].

From a biological and physiological point of view, some authors describe COVID-19 infection as a turning point that “collapses the house of cards” in frail and vulnerable patients [6]. Others, from a political and sociological perspective, suggest that COVID-19 collapsed the long-term care system due to a mix of structural and political/socio-cultural factors [10], and they shed light on the historical under-resourcing of long-term care and its marginalisation from the political agenda [11]. In many cases, this has often resulted in a series of structural characteristics in care homes that may have contributed to the severity of the impact of the pandemic in these facilities, such as excessively high numbers of residents, high occupancy and overcrowding [12], inappropriate design standards of the facilities, and a high prevalence of private for-profit homes and/or chain ownerships [13], as well as a low presence of qualified health professionals [14] and growing job insecurity in the long-term care home sector [15].

The first confirmed COVID-19 case in Spain was reported on 31 January 2020, and the first fatality in a care home was confirmed on 3 March. After that, the mortality figures in care homes spiked dramatically and most Spanish care homes were overwhelmed. Indeed, official data show that 19,835 confirmed or suspected COVID-19 deaths occurred among care home residents between 14 March and 22 June 2020. This figure accounts for around 70% of the total confirmed deaths from COVID-19 during that period [16].

### 1.1. Health Care and Long-Term Care in Spain: A Background

Spain has a universal health care system with both public and private providers, and the whole population, regardless of individual economic status, is assured free access to health care. The provision of health care services is highly decentralised and falls under the jurisdiction of the 17 Autonomous Communities (hereinafter ACs), which are geographical, cultural, and political regions with their own independent health and social services. In recent years, the variation per capita expenditure across the ACs has increased. The system has grown substantially since the late 1970s, when citizens’ rights to health care were recognised. Despite the increase in the demand for care because of demographic trends, the Spanish public administrations’ response to the 2008 economic crisis was to cut public health spending and adopt a market-oriented and privatising approach to the management of the health system. The public health response to the lethal impact of COVID-19 in care homes needs to be understood from within the context of a weakened Spanish public health sector.

The responsibility for social care in Spain also lies with the 17 ACs. Since 1978 the AC governments have developed their public social services systems [17] according to their own political tradition and budgeting [18,19]. This has resulted in regional differences in terms of political priorities and financial commitments, service coverage and accessibility [20].

Care homes in Spain are part of long-term care (LTC) facilities that can be either publicly or privately owned (for-profit or non-profit), and their management may also be public or private. Public providers are organisations in which public authorities (AC governments or municipalities) are the direct managers or have the power to appoint management. Private providers usually offer both private services and services outsourced by the public sector (often both kinds within the same facility). The latter refers to any public service offered by a private provider that has signed agreements with a public administration [21].

Since 2007, LTC in Spain has been regulated by the National Act 39/2006 on the Promotion of Personal Autonomy and Care for Dependent Persons (hereinafter the DL). The DL instituted the right to receive public and universal LTC for the first time ever, and it was designed to democratise and improve accessibility to LTC services by reorganising their provision. Furthermore, the DL consolidated the participation of the private sector in LTC, both through its inclusion in the public network and through private initiatives funded by users. The Spanish LTC scenario became more complex following the application of austerity policies, social cutbacks and the further privatisation of LTC public services as a result of the 2008 crisis. The funding of services and quality of care provided in care homes were affected negatively. Therefore, the COVID-19 pandemic struck an underdeveloped and highly privatised LTC system in Spain.

### 1.2. Research Goals

The goal of this study was twofold. First, we aimed to describe the impact of COVID-19 in Spanish care homes during the first wave of the pandemic (March–June 2020). Second, we analysed the regional variation in COVID-19 deaths among care home residents, considering those factors identified in the literature that may contribute to explaining the high death toll in those facilities.

## 2. Methods

We conducted methodological triangulation using quantitative and qualitative data and three analytical procedures: a documentary analysis, a quantitative descriptive analysis, and an ecological analysis with a regression model.

### 2.1. Documentary Analysis

The documentary analysis was carried out between April 2020 and December 2021. It included a variety of documents: (1) scientific literature, (2) reports by several national and international agencies, (3) press releases from Spanish administrations, and (4) transcriptions of the health minister’s public statements. This analysis aimed to identify the main factors influencing the high mortality rates in care homes. To this end, we carried out a narrative review with a qualitative approach in order to understand the situation at the beginning of the pandemic. The search strategy applied to the scientific literature review included the keywords “COVID-19”, “mortality”, “care home * OR nursing home *”, “long-term care”, and “older people”. The search limits were publication type (original articles and reviews) and language (English and Spanish). We ran the searches in the Scopus and PubMed databases. We also used a snowball strategy to review the references cited in the retrieved articles.

After the first screening by title and abstract, we analysed 55 journal articles. These articles discussed the impact of the pandemic on the care home systems of various Global North countries, such as Belgium, Canada, England, France, Italy, Scotland, Singapore, Spain, and the United States. Their main purpose was to describe the key factors underlying care home COVID-19 cases and mortality within a single country or region. We also analysed 36 national and international health and social reports published by the Spanish Ministry of Social Rights; the Spanish Institute of Older Adults; the Spanish Institute of Statistics; the Spanish Centre of Epidemiology; Spanish regional or AC governments; Spanish and international non-profit organisations; and, among other sources, Spanish and international research institutions. The review identified several potential factors related to higher COVID-19 incidence and mortality rates in care homes during the first wave of the pandemic. For the ecological analysis we selected those that were applicable to the Spanish context and that we had available public data.

### 2.2. Quantitative Analysis

The quantitative analysis was intended to reveal the impact of COVID-19 in care homes and its relationship with some relevant characteristics of care homes in each of the 17 ACs. We obtained our data on care home COVID-19 mortality from the following primary and secondary sources: (1) Spanish central government official reports published by the Health Alerts and Emergencies Coordination Centre, the Spanish National Statistics Institute (INE), the Daily Mortality Surveillance System, the Spanish Institute for the Elderly and Social Services (IMSERSO), and the Spanish National Research Council (CSIC); (2) AC official sources, such as information published through their websites or transparency portals, press releases, and epidemiological reports; and (3) information that the researchers actively requested from the Spanish central government and AC transparency portals.

We used five different indicators of COVID-19 mortality among care home residents: (1) the percentage of total COVID-19-related deaths accounted for by care home residents; (2) the percentage of the total number of care home residents who died from COVID-19; (3) the percentage of excess deaths; (4) the percentage of COVID-19 deaths certified as having occurred in a care home; and (5) the excess deaths among care recipients who occupied publicly funded beds in care homes, which was the most robust of the five indicators in the Spanish context. Indicators based on case identification are less reliable due to the scarceness of testing resources in the care homes and the case definition used during most of the first wave (only cases confirmed by PCR tests). In addition, the lack of publicly available data on care homes makes it difficult to obtain exact denominators for some indicators (e.g., care home population in each AC).

Furthermore, we used a descriptive ecological approximation to depict the trends in some of the explanatory factors identified in the scientific literature and technical reports as relevant when explaining AC variability in COVID-19 mortality rates in care homes. In order to enhance the assessment of the association between variables in the multiple group analysis [22,23], we complemented the ecological analysis with a linear regression model using Jamovi (version 1.6.23.0). Each AC was used as a unit of observation. The indicator “excess deaths among care recipients who occupied publicly-funded beds in a care home” was selected as the outcome variable represented on the *y*-axis. This indicator shows excess deaths among the 1,121,520 (2020) recipients of publicly funded LTC who occupied a bed in a care home, a figure which amounts to 72.6% of the total care home resident population while omitting privately funded bed occupants. Intergroup variability was examined for the main factors identified in the literature review (*x*-axis) for which there was public data available. The factors used as independent variables were (1) cumulative incidence, (2) number of care home beds in each AC, (3) care home size, and (4) type of ownership. Both dependent and independent variables were measured for groups.

We first constructed a regression model with all independent variables and the *p*-value being established under <0.05. We then excluded the non-significant variables. We performed a Shapiro–Wilk normality test, necessary for linear model construction with a sample n < 30, which indicated the normality of the data (*p*-value = 0.592). We also conducted tests to discard collinearity between the significant independent variables, obtaining a VIF of 1.07 and a Durbin and Watson statistic of 1.90. In parallel, we created a correlation matrix with all care home-related independent variables to detect possible relationships between them.

## 3. Results

### 3.1. Mortality Indicators in Spain

Table 1 shows the data provided by the five indicators we used to quantify COVID-19 deaths among care home residents in each of the Spanish ACs.

**Figure 1:**
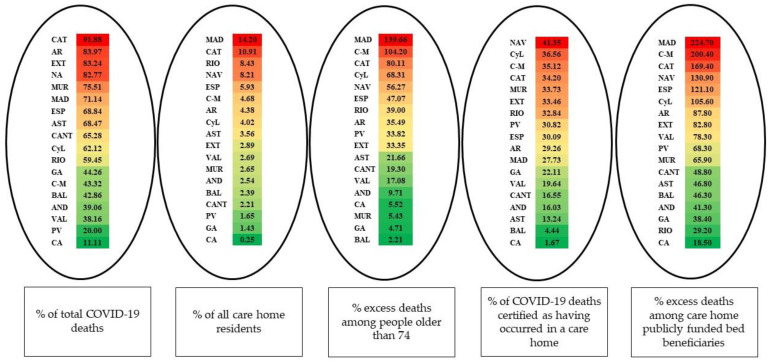
Autonomous Communities ranked by care home COVID-19 mortality indicators. Source: Authors’ work using the following data: the percentages of total COVID-19 deaths and percentages of all care home residents correspond to the period comprising 15 March to 21 June 2020. As regard to care home population the date varies depending on the source. For care home population and beds ACs provided data ranging from June 2020 to January 2021, whereas IMSERSO data [24] for Andalucía is from December 2019, and CSIC data [25] for Canarias and Castilla-La Mancha is from September 2020. These two indicators are based on public data provided by the Spanish central government and AC administrations and their responses to the authors’ requests for information. The figures for excess deaths among people older than 74 were provided by the Daily Mortality Surveillance System (MoMo) and cover 14 March to 23 June. The percentages of COVID-19 deaths in care homes come from INE databases [26] and refer to March, April, and May 2020. The excess deaths indicator among publicly funded beneficiaries refers to March, April, May and June, and the data comes from IMSERSO [27].

As shown in Figure 1, after the first wave of the pandemic Catalunya, Aragón, Extremadura, Navarra, Murcia, and Madrid were the ACs with the highest percentages of deceased care home residents among total COVID-19 deaths, ranging from 71.14% in Madrid to 91.88% in Catalunya. This stands in sharp contrast to Canarias and País Vasco, where the percentages with regard to the first indicator were 11.11% and 20% respectively.

Madrid, Catalunya, La Rioja, and Navarra were the four ACs that witnessed the highest impact of COVID-19 mortality on their care home resident population, with Madrid and Catalunya presenting rampant percentages of 14.20% and 10.91%, respectively. At the other extreme, País Vasco, Galicia, and Canarias presented comparatively low percentages (1.65%, 1.43%, and 0.35%), all of them below 2% and well under the Spanish average of 5.93%.

Madrid, Castilla-La Mancha, and Catalunya were the three ACs with the highest percentages of excess deaths among people older than 74, amounting to 139.44%, 104.20% and 80.11%, respectively, whereas the figures for other ACs such as Canarias, Murcia, Galicia, and Baleares were comparatively low, totalling 5.52%, 5.43%, 4.71%, and 2.21%, respectively. The Spanish average was 47.07%. However, this indicator only offers data on the mortality rate that may be attributable to COVID-19 among older adults who lived in care homes and those who did not.

The Spanish average percentage of COVID-19 deaths in care homes in comparison with all COVID-19 deaths was 30.09%. This means that over 30% of the total deceased were not admitted to a hospital or sent to their family home, among other destinations. This indicator in Navarra, Castilla y León, Castilla-La Mancha, Catalunya, and Murcia attains values that range from 33.73% (Murcia) to 41.35% (Navarra). At the other extreme, 1.67%, 4.44%, and 12.24% of total COVID-19 deaths occurred in care homes in Canarias, Baleares and Asturias, respectively.

Finally, the percentage of excess deaths among care home publicly funded bed beneficiaries in Spain was 121.10%. The figures were higher in Madrid, Castilla-La Mancha, Catalunya, and Navarra, reaching 224.70%, 200.40%, 169.40%, and 130.90%, respectively. There were, however, several ACs that performed much better, with outcomes well under half the Spanish average: Canarias (18.50%), La Rioja (29.20%), Galicia (38.40%), Andalucía (41.30%), Baleares (46.30%), Asturias (48.80%), and Cantabria (48.80%).

### 3.2. Factors Underlying COVID-19 Care Home Mortality in Spain

Among all the factors identified by the literature that made it more likely for COVID-19 outbreaks and deaths to occur in LTC residential facilities, we analysed the following: (1) the COVID-19 cumulative incidence per 100,000 inhabitants in the surrounding area of care homes; (2) the number of care home beds in each AC; (3) average care home size in each AC; and (4) the percentage of private care homes in each AC (type of ownership). We selected these factors according to the relevance assigned to them in the literature and the (scarce) available data on care home characteristics in Spain. Table 2 displays the COVID-19 mortality indicator of excess deaths among care home publicly funded bed beneficiaries and the figures for these four factors in each of the ACs.

### 3.3. Cumulative Incidence in the Surrounding Area of Care Homes

**Figure 2:**
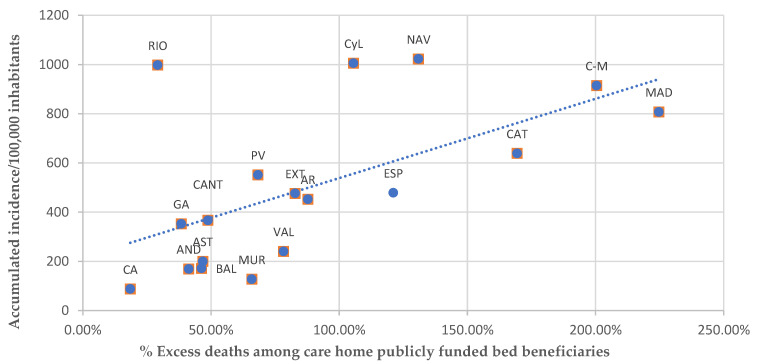
Excess mortality among care home publicly funded bed beneficiaries and accumulated incidence/100,000 inhabitants in each Autonomous Community. Source: Authors’ work using data from the Centro Nacional de Epidemiología [28] and IMSERSO [27].

Several studies point out a positive relationship between the incidence of the COVID-19 in a particular geographical area and its impact on the care homes within that area [13,29,30]. The higher the incidence in the region surrounding a care home the higher the probability of introduction of COVID-19 by visitors or staff [12]. This also means more likelihood of infected professionals [31] and higher rates of positive and lethal cases among residents. Figure 2 displays the relationship between the cumulative incidence and excess mortality among care home residents in Spain.

The cumulative incidence per 100,000 inhabitants was 478.70, and there were seven ACs that surpassed this average, some of them more than doubling it. In agreement with the literature, the general trend seems to be that the higher the incidence of COVID-19 in a particular AC, the higher the number of excess deaths in its care homes. There were some exceptions. For example, while the cumulative incidence in La Rioja was among the highest in the country (997.80), its care home excess death toll was the second lowest (29.20%). Also, while the cumulative incidence in Castilla y León almost doubled the national average (1005.30), care home excess mortality there was lower than the national average (105.60%). This factor needs to be further analysed using disaggregated data on COVID-19 mortality in each care home and on local cumulative incidence. However, this information is not publicly available.

### 3.4. Number of Care Home Beds in the AC

**Figure 3:**
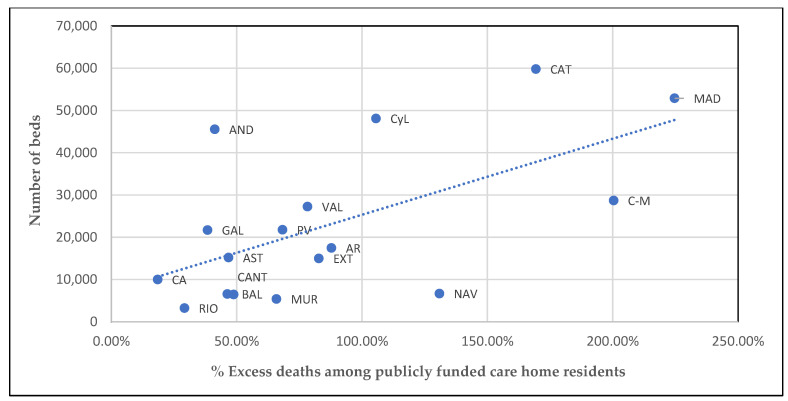
Excess deaths among care home publicly funded bed beneficiaries and number of beds in each AC. Source: Authors’ work using Spanish central government and AC administration responses to the authors’ requests for information (Aragón, Catalunya, Extremadura) and data from IMSERSO (the rest) [26,27].

While there is scarce previous evidence on this factor, a few reports suggest an association between the number of available care home beds in a particular region and the infection and death of elderly people in its care homes [32,33]. Figure 3 shows the relationship between excess deaths in care homes and the absolute number of care home beds in each Spanish AC.

In Spain in 2020 there were a total of 391,663 care home beds. The number of beds in Catalunya, Madrid, Castilla y León, and Andalucía was over 40,000 in each AC. This variable seems to be relevant as an explanatory factor since there is a positive relationship where the higher the number of care home beds in the AC, the higher the excess mortality. This is clear in the cases of Madrid and Catalunya, with high excess death rates and high numbers of care home beds, and Canarias and La Rioja, with comparatively low figures for both variables. Nevertheless, there are outliers. Excess deaths in Andalucía were far below the national average whereas its care home bed numbers were amongst the highest in the country. At the other extreme, Navarra had fewer than 7000 beds and its care home excess mortality was the fourth highest in the country.

### 3.5. Care Home Size

**Figure 4:**
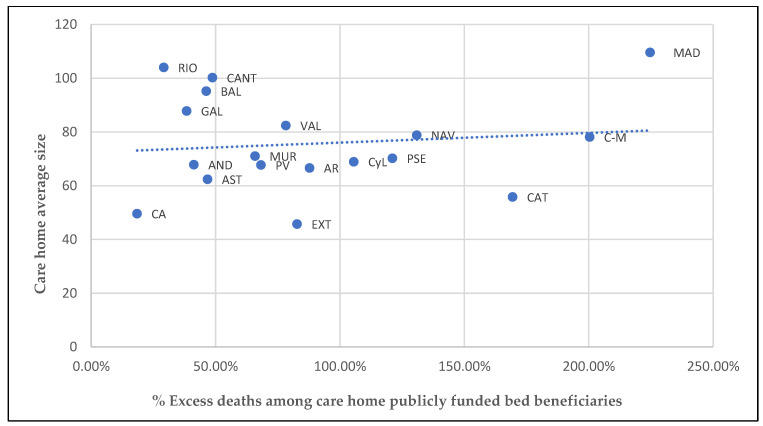
Excess deaths among care home publicly funded bed beneficiaries and average number of beds in care homes in each AC. Source: Authors’ work using data from IMSERSO [24,27].

A vast number of research papers and technical reports have examined the influence of the size of care homes on mortality. Some report a positive correlation between the number of residents and the probability of COVID-19 outbreaks [12,34,35]. Others hint that while larger homes were more likely to have outbreaks, outbreaks at small facilities affected proportionately more patients [36,37] and resulted in higher mortality. Studies that focused on the pandemic in Spain suggest that there were more fatalities in ACs with care homes of a higher average size [15,31,32,38]. The higher the number of residents and workers, the higher the number of outside and inside contacts, and, therefore, the higher the risk of infection. Nonetheless, the Spanish care home data displayed in Figure 4 does not support this connection. 

The average care home size in Spain was 70.20 beds in 2020. ACs with an average of more than 70 beds in their care homes, such as Madrid, Castilla-La Mancha, and Navarra, had a higher death toll in these facilities, whereas ACs with a mean number of fewer than 50 beds, such as Canarias and Extremadura, experienced fewer deaths. However, other ACs with a high care home average size (Rioja, Cantabria, Baleares, and Galicia) had a low number of excess deaths, whereas in ACs such as Catalunya, with a high proportion of excess deaths, the average size of care homes was relatively low. Overall, Figure 4 does not show any clear relationship between the two variables. Even so, this factor needs further analysis using disaggregated data (on each care home), which is currently not available in Spain.

### 3.6. Ownership

**Figure 5:**
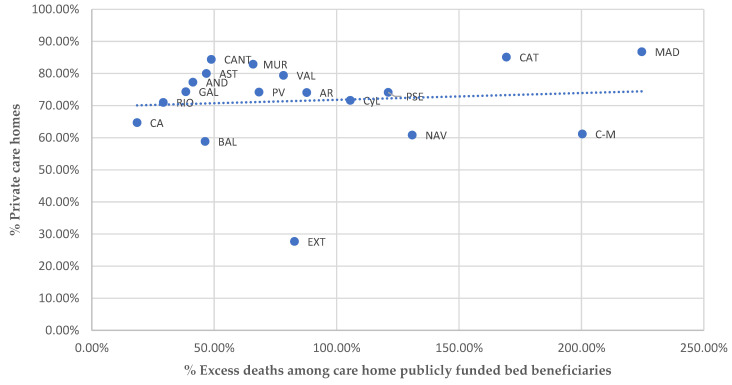
Excess deaths among care home publicly funded bed beneficiaries and type of ownership. Source: Authors’ work using data from IMSERSO [24,27].

A great deal of research has analysed the impact of the type of care home ownership on COVID-19 outbreaks and mortality and, more specifically, whether private and for-profit status increased their likelihood [38,39,40]. Whereas some authors conclude that the type of ownership may have had a relevant influence [15], others argue that this association is mediated and largely explained by other factors, such as the type of private ownership (big chains) or architectural design [13], the larger size of private homes, and their tendency to be located in urban and suburban areas [29]. Although Figure 5 shows the high proportion of private ownership in most ACs, it does not reveal any clear relationship between the type of ownership and care home excess deaths. 

The percentage of private care homes across the country is 74.12% and only in one AC, Extremadura (27.69%), is it under 50%. In some ACs, such as Catalunya, Castilla-La Mancha, and Madrid, the degree of privatisation and excess mortality figures are both very high. However, in other ACs, such as Cantabria and Murcia, excess death numbers are below the average while the proportion of private care homes is still very high. Yet again, disaggregated data on the type of care home ownership and management in Spain is not publicly available.

### 3.7. Factor Associations

Finally, we assessed the associations among these four explanatory factors by building a regression model. We show the model summary in Table 3, where the AC accumulated incidence and the total number of care home beds in the AC are the regressors. Both factors are part of the model and its expression is as follows: Excess deaths = 6.66113 + 0.08643 accumulated incidence/100,000 inhabitants + 0.00160 total number of care home beds. This is the model that best fits the data after performing ANOVA analysis and reducing the independent variables according to their significance.

In Table 4 we can see that this model explains 51.3% (R^2^ adjusted) of the variance of the excess mortality, by AC, in publicly funded care home beds. In other words, the accumulated incidence and total number of care home beds explain more than half of excess deaths among publicly funded care home residents.

We also conducted an analysis of the care-home-related independent variables (Table 5) and found a moderate positive correlation between care home average size and percentage of private care homes (0.368 Pearson’s R) and between total number of care home beds and private care homes (0.337 Pearson’s R). This suggests that the higher the percentage of private care homes in ACs, the higher their average size. In addition, the higher the number of total care home beds in each AC, the higher its percentage of private care homes. We also found a moderate negative relationship between the total number of care home beds and care home average size (−0.102). Therefore, the variable with the most ability to explain the characteristics of care homes across Spanish AC care homes is the type of ownership. Having said this, since the *p*-value is higher than 0.05 in all these cases, these relationships do not offer statistical evidence. Additional variables and disaggregated data are needed to confirm their significance. However, this data is not publicly available at the moment.

## 4. Discussion

As shown in the results, the impact of the pandemic in Spanish care homes varied greatly across the ACs. Regarding the factors that may explain this variation, our statistical analysis points to a relationship between the excess deaths in care homes and (1) COVID-19 accumulated incidence in ACs, and (2) the number of beds in each AC. Although there is scarce literature on the role the number of care home beds in a particular region may have played in the pandemic, our results for accumulated incidence are aligned with both the scientific and technical literature in that the excess mortality among older people who resided in care homes could be explained by the difficulty in preventing the virus from entering the facilities, spreading, and causing deaths.

On the other hand, the trend toward privatisation in the Spanish LTC sector may be relevant when analysing the impact of COVID-19 in care homes. During the past decade, the emphasis on financial discipline in the context of European demographic ageing has often portrayed ageing as a financial burden and a threat to fiscal sustainability. Despite the increase in the demand for care as a result of demographic trends, Spanish public administrations have cut public health spending and adopted a market-oriented approach, including the creeping privatisation of healthcare. After the explosion of the 2008 economic crisis, the Spanish central government and several ACs introduced austerity measures that resulted in a significant reduction in health care spending. Consequently, the response of Spanish public administrations to the lethal impact of COVID-19 in care homes was framed within the context of a weakened Spanish public health sector. A deteriorated and overloaded infrastructure faced serious difficulties when responding to the effects of the pandemic and providing care to older adults living in care homes [41,42].

The COVID-19 pandemic also struck an underdeveloped and highly privatised LTC system in Spain. The weakness of the LTC sector dates back to before the 2008 economic crisis. The growth of the neoliberal model since the 1970s and the historically poor development of the Spanish welfare state led to the emergence of an enormous care market. During the 1970s and 1980s, the first business-oriented private care homes emerged. Starting in the late 1980s, and to a greater degree in the late 1990s, there was a transfer of public funds and care responsibilities to private companies through tenders for a huge number of care homes and their complete concession. In 2007, with the approval of the aforementioned DL, the funding of LTC was to be split between the Spanish central government and the ACs and Municipalities. However, imbalances in level of accessibility, quality, and regional distribution persisted [43]. Over the years, the tensions between different administrative levels, insufficient funding, inadequate staff-to-resident ratios, and the lack of an accurate evaluation of resources led to inefficient implementation of services [44].

The DL’s inclusion of the private sector in the public network of LTC providers consolidated the already existing outsourcing of care services to private companies [45,46]. The increase in LTC public spending benefitted private initiatives through the purchase or partial funding of public beds in private care homes and the outsourcing of publicly owned care homes to private companies. In a context of rising demand for LTC, the DL turned the Spanish care home sector into an attractive market for Spanish and international investors and accelerated its privatisation. This scenario became more complex after the application of austerity policies, social cutbacks, and the further privatisation of LTC public services resulting from the 2008 crisis [47,48]. Funding of services was affected negatively, the quality of care provided in care homes declined, and public administrations adopted a more permissive approach to staff qualifications, staff-to-resident ratios, equipment, and infrastructure standard compliance [49].

Although our ecological analysis does not show any clear patterns when it comes to the impact of care home size and type of ownership, it suggests that the number of private care homes can be an explanatory variable of interest when drawing the map of care homes in Spain. Therefore, due to its relationship with the number of care home beds in each AC, and given the recent history of the Spanish health care and care home systems, the privatisation process and the proportion of private care homes could also explain the lethal impact of the pandemic in those facilities during the first wave. There is a lack of publicly available disaggregated data for each individual care home with regard to COVID-19 mortality and the independent variables. Given that the literature suggests that one of the main factors influencing COVID-19 care home mortality has been the type of ownership [15], its role during the pandemic could be further assessed if the aforesaid disaggregated data was available.

In Spain, there is an apparently paradoxical trend in care home ownership and the proportion of public and private beds in care homes. Whereas the percentage of privately owned care homes slightly diminished between 2006 and 2020, the proportion of private beds in care homes increased. This may be explained by the fact that private care homes in Spain tend to be larger and publicly owned care homes smaller [25]—24.5% of the former have more than 100 beds compared with 18.05% of the latter. Care home size is also a highly discussed factor in the literature because of the likely positive correlation between the number of residents, outside and inside contacts, and the probability of COVID-19 outbreaks [12,34,35]. Furthermore, our results show a moderate positive correlation between average care home size and the percentage of private care homes.

Besides homes and beds, the distinction between care home ownership and management may also be relevant since the massive outsourcing of care services to private companies in recent times has produced a LTC scenario in Spain where publicly owned care homes are often privately managed. In fact, the number of care homes that are both publicly owned and run is insignificant (only 11.4% of care homes and 13.1% of beds). Once again, the unavailability of public disaggregated data on type of care home management prevented us from further examining this relationship.

### Data Limitations

The main limitation of this study was the lack of available and reliable data, which limited the accuracy of impact assessment and complicated regional comparisons [40,50]. The Spanish central government did not release official nationwide data on COVID-19 infections and deaths in LTC facilities during the first wave. The only available information came from the media and AC governments. The reliability of the data was, in turn, undermined by several factors such as case definition (modified several times) and data collection and reporting (testing availability during the period under study and whether official information included suspected COVID-19 cases or not). Since Spain had a poor testing capacity during the first wave, deaths occurring outside hospitals were omitted from the official statistics [51]. All these factors may have contributed to the variation in the estimated number of COVID-19 cases and deaths in the ACs.

The non-existence of an up-to-date database on the total elderly population living in care homes in Spain constituted another limitation when constructing the indicators. Furthermore, there is no updated data that describes the real conditions in which care home residents live. Despite all these limitations, this article sheds further light on what happened in Spanish care homes and helps to expose the opaque management of the epidemiological surveillance cycle in this context.

In turn, these limitations on data reliability and accessibility affected the scope of the ecological approach. Each AC was used as a unit of observation and both dependent and independent variables were measured as aggregate, even though some of them were individual-level variables (average size and ownership) and averages can misrepresent the real circumstances. However, as noted repeatedly throughout this article, disaggregated data at the care home level is not publicly available in Spain.

Finally, the indicator used for the ecological analysis (excess deaths among care recipients who occupied publicly funded beds in care homes) is not without limitations since it omitted privately funded bed occupants. We still consider it the most robust available indicator since it is not affected by the scarceness of PCR testing in care homes. Furthermore, it takes into account the “collateral” deaths provoked by the lack of an appropriate response from an overstressed health system. 

## 5. Conclusions

The first wave of the COVID-19 pandemic had an enormous impact on older adults living in Spanish care homes. According to the Spanish central government, almost 20,000 older adults died in these facilities. This study puts some order into the dispersed and incomplete epidemiological data and sheds light on the real impact of the pandemic on care home residents.

The variation in mortality indicators across Spain is especially relevant because the social services are the responsibility of the AC governments. Numerous factors may explain this variability, but the clearest trend reflected by our data is that the higher the cumulative incidence and the number of care home beds in the surrounding area (AC), the higher the COVID-19 mortality in care homes. This is a crucial reminder of the importance of community-based preventive measures and social policies.

Further analysis, using individual care home disaggregated data, needs to be conducted on the impact of care home size and type of ownership in order to assess the role that these variables may have played during the pandemic. Individual care home data, if linked to COVID-19 deaths in care homes, could be crucial to understanding the factors that may have contributed to the spread of COVID-19 within those facilities and the existing variation across ACs. In fact, our results show that care home disaggregated data on size, type of ownership, and management may be able to account for at least part of what happened in Spanish care homes and the variation among ACs. Data availability is crucial to understanding the causes behind the spread of COVID-19 within care homes. Without data, our ability to learn the lessons needed to anticipate future actions and improve public policies is limited. This requires transparency policies and the public dissemination of epidemiological data, as well as proper accountability on the part of public administrations.

Furthermore, qualitative research aimed at exploring the experiences of older adults, care home professionals and health and social decision-makers, among other stakeholders, could shed light on additional factors relevant to the management of the crisis. The COVID-19 pandemic took place within a context of an underdeveloped and highly privatised LTC system with a limited ability to protect care home residents. Although the factors and constraints we have discussed in this article were intrinsic to the Spanish pre-pandemic LTC sector, the crisis has brought to light its fragility and these factors have exacerbated the consequences of the pandemic, with a death toll running into tens of thousands. Further research on these issues is crucial to rethinking the long-term care system in Spain in order to improve its preparedness for future crises and prioritise older adults’ needs. 

## Figures and Tables

**Table 1:** Care home COVID-19 mortality indicators in Spain disaggregated by Autonomous Communities.

	Number of COVID-19 Deaths in Care Homes ^a,b^	% of Total COVID-19 Deaths ^a,b^	% of All Care Home Residents ^a,b^	% Excess Deaths among People Older Than 74 ^c,d^	% of COVID-19 Deaths Certified as Having Occurred in a Care Home ^e,f^	% Excess Deaths among Publicly Funded Care Home Residents ^g,h^
Andalucía	557	39.06%	2.54%	9.71%	16.03%	41.30%
Aragón	765	83.97%	4.38%	35.49%	29.26%	87.80%
Asturias	228	68.47%	3.56%	21.66%	13.24%	46.80%
Baleares	96	42.86%	2.39%	2.21%	4.44%	46.30%
Canarias	18	11.11%	0.25%	5.52%	1.67%	18.50%
Cantabria	141	65.28%	2.21%	19.30%	16.55%	48.80%
Castilla y León	1725	62.12%	4.02%	68.31%	36.56%	105.60%
Castilla-La Mancha	1309	43.32%	4.68%	104.20%	35.12%	200.40%
Catalunya	5206	91.88%	10.91%	80.11%	34,20%	169.40%
Comunidad Valenciana	546	38.16%	2.69%	17.08%	19.64%	78.30%
Extremadura	432	83.24%	2.89%	33.35%	33.46%	82.80%
Galicia	274	44.26%	1.43%	4.71%	22.11%	38.40%
Comunidad de Madrid	5987	71.14%	14.20%	139.66%	27.73%	224.70%
Murcia	111	75.51%	2.65%	5.43%	33.73%	65.90%
Navarra	437	82.77%	8.21%	56.27%	41.35%	130.90%
País Vasco	311	20.00%	1.65%	33.82%	30.82%	68.30%
La Rioja	217	59.45%	8.43%	39.00%	32.84%	29.20%
Total Spain	18,360	68.84%	5.93%	47.07%	30.09%	121.10%

Source: Authors’ work using public data available from: ^a^ the Spanish central government and AC administrations and their responses to the authors’ requests for information; ^b^ from 15 March to 21 June 2020. As regards care home population the date varies depending on the source: for care home population and beds, ACs provided data ranging from June 2020 to January 2021, whereas IMSERSO data for Andalucía [24] is from December 2019 and CSIC data for Canarias and Castilla-La Mancha [25] is from September 2020; ^c^ Daily Mortality Surveillance; ^d^ from 14 March to 23 June; ^e^ INE [26]; ^f^ March, April, and May 2020; ^g)^ IMSERSO [27]; ^h^ March, April, May, June 2020.

**Table 2:** Excess deaths among care home publicly funded bed beneficiaries and characteristics of care homes in Spain disaggregated by Autonomous Communities.

	% Excess Deaths among Publicly Funded Care Home Residents ^a,b^	Accumulated Incidence/100,000 Inhabitants ^c,d^	Total Number of Care Home Beds ^e,f^	Care Home Average Size ^g,h^	% Private Care Homes ^g,h^
Andalucía	41.30%	168.90	45,543	67.80	77.29%
Aragón	87.80%	452.40	17,462	66.60	74.05%
Asturias	46.80%	199.80	15,204	62.40	80.00%
Baleares	46.30%	171.60	6573	95.20	58.82%
Canarias	18.50%	87.60	9994	49.60	64.68%
Cantabria	48.80%	366.70	6444	100.20	84.38%
Castilla y León	105.60%	1005.30	48,089	68.90	71.63%
Castilla-La Mancha	200.40%	914.40	28,695	78.10	61.16%
Catalunya	169.40%	638.90	59,792	55.80	85.10%
Comunidad Valenciana	78.30%	240.30	27,248	82.40	79.39%
Extremadura	82.80%	476.20	14,974	45.70	27.69%
Galicia	38.40%	352.50	21,704	87.80	74.32%
Comunidad de Madrid	224.70%	807.20	52,882	109.60	86.76%
Murcia	65.90%	127.30	5395	71.00	82.89%
Navarra	130.90%	1022.90	6664	78.80	60.81%
País Vasco	68.30%	551.40	21,765	67.70	74.19%
La Rioja	29.20%	997.80	3235	104.00	70.97%
Total Spain	121.10%	478.70	391,663	70.20*	74.12%

Source: Authors’ work using data from: ^a^ IMSERSO [27]; ^b^ March, April, May, June 2020; ^c^ Centro Nacional de Epidemiología [28]; ^d^ from 15 March to 21 June 2020; ^e^ Spanish central government and AC administration responses to the authors’ requests for information (Aragón, Catalunya, Extremadura) and IMSERSO [24] (the rest of the ACs); ^f^ from June 2020 to January 2021 (Aragón, Catalunya, Extremadura) and December 2019 (the rest of the ACs); ^g^ IMSERSO [24]; ^h^ December 2019. This table excludes the autonomous cities of Ceuta and Melilla, which were not analysed in our study. Since they have very small populations, they do not have a significant impact on the total figures for Spain.

**Table 3:** Linear regression model after eliminating non-significant independent variables.

Model Coefficients–Excess Deaths among Publicly Funded Care Home Residents				
Predictor	Estimate	SE	t	*p*
Intercept	6.66113	21.3295	0.312	0.759
Accumulated incidence/100,000 inhabitants	0.08643	0.0328	2.639	0.019
Total number of care home beds	0.00160	6.04 × 10^−4^	2.659	0.019

Source: Authors’ work using Jamovi (Jamovi: R-project interface, Sydney, Australia).

**Table 4:** R and R-squared of the model.

Model Fit Measures			
Model	R	R^2^	Adjusted R^2^
1	0.757	0.574	0.513

Source: Authors’ work using Jamovi (Jamovi: R-project interface, Sydney, Australia).

**Table 5:** Correlation matrix between home-related independent variables.

		Private Care Homes	Care Home Average Size	Total Number of Care Home Beds
**Private Care Homes**	Pearson’s r	-	-	-
	*p*-value	-	-	-
**Care Home Average Size**	Pearson’s r	0.368	-	-
	*p*-value	0.147	-	-
**Total Number of Care Home Beds**	Pearson’s r	0.337	−0.102	-
	*p*-value	0.185	0.698	-

Source: Authors’ work using Jamovi (Jamovi: R-project interface, Sydney, Australia).

## Data Availability

The data presented in this study are available in the article.
